# The diminishing returns of distance: Computational analysis of approach-avoidance behavior under threat of predation

**DOI:** 10.1371/journal.pone.0360036

**Published:** 2026-10-28

**Authors:** Jonathon R. Howlett, Alan N. Simmons, Katia M. Harlé, Daniel M. Stout

**Affiliations:** 1 VA San Diego Healthcare System, San Diego, California, United States of America; 2 Department of Psychiatry, University of California San Diego, San Diego, California, United States of America; University of Toyama: Toyama Daigaku, JAPAN

## Abstract

Foraging under threat of predation is a paradigmatic scenario involving the interplay between approach and avoidance motivation across species. Previous foraging paradigms in humans have revealed aspects of approach-avoidance behavior and its neural underpinnings, but have generally involved simple choices such as a binary decision to stay or flee. Here, we present results from 40 adult participants who completed the Weighing Opportunity and Loss in Foraging (WOLF) task, a novel two-dimensional foraging context with threat of predation in which participants choose from five possible actions at each turn. We fit computational models in which choices depend on the subjective utility of each option, which itself depends on approach motivation (resource richness) and avoidance motivation (predator distance). Model comparison revealed that an inverse power law model, in which the marginal subjective utility of additional distance from the wolf diminished with wolf distance, outperformed a linear model. Model parameters explained more than 75% of the variance in task performance, mediated by the average resource richness of choices (for the approach-related parameter) and the number of deaths on the task (for the avoidance-related parameters). An exploratory analysis found that self-reported anxious arousal was associated with a more linear relationship between wolf distance and avoidance motivation. The results demonstrate that a behaviorally rich foraging paradigm in which participants select from multiple options at each turn can enable explicit modeling of subjective utilities in the interplay between approach and avoidance motivation. Future work can examine neural underpinnings of complex approach-avoidance behavior and their alteration in mental health disorders.

## Introduction

Approaching rewarding stimuli and avoiding threats are core behaviors that support survival across species [1] and are supported by evolutionarily conserved neural mechanisms [2]. Ethological investigations of approach and avoidance behavior have frequently focused on foraging contexts, in which animals must approach food while avoiding predators. A key concept in this literature is the flight initiation distance (FID), or the distance between a prey animal and approaching predator at which the prey animal begins an escape behavior to avoid harm [3]. Optimal behavior depends on a tradeoff between the risk of predation if the FID is too small and the loss of foraging opportunities if the FID is too large [4,5], illustrating the importance of balancing competing approach-avoidance imperatives to promote survival.

Approach-avoidance behavior has been extensively investigated in humans and is thought to be particularly relevant to psychiatric disorders such as mood and anxiety disorders [6–8]. Some studies have examined active avoidance behavior under threat of virtual predators and showed shifts in neural activity (e.g., from ventromedial prefrontal cortex to periaqueductal gray) as a predator moves closer [3,9]. This is thought to represent a transition from a state of cognitive anxiety to a state of acute fear. More recently, an FID behavioral paradigm has been developed, in which subjects decide when to flee from a virtual predator in a one-dimensional environment and gain reward (money) by delaying flight as long as possible. The FID task has enabled characterization of different neural circuits underlying slow and fast escape decisions [10].

While the FID paradigm and similar tasks have generated significant insight into human approach-avoidance performance in simple foraging contexts, the computational underpinnings of such behavior in richer and more complex contexts have been less explored. For example, approach-avoidance tasks typically consist of a decision between only two options, i.e., an approach option and an avoidance option [8] or allow the subject to move along a one-dimensional continuum between two stimuli [2]. Similarly, the FID paradigm consists of a one-dimensional environment in which the main behavior is a categorical decision to flee and the behavioral outcome is the distance from the predator at which this flight occurs (i.e., the FID). By contrast, in real behavioral contexts, humans and other animals often have more than two concurrent behavioral options and may balance the influence of threats and rewards on decisions in a graded rather than binary (stay or flee) manner.

Several computational modeling approaches have been used to elucidate the basic underlying mechanisms of approach-avoidance behavior and their potential disruption in psychiatric disorders, though they are typically limited to binary or one-dimensional paradigms [11]. Drift-diffusion models, which incorporate both choices and reaction times to examine the process of accumulating evidence for different choices, have been applied to tasks in which a binary choice is made between approach and avoidance options. This approach has yielded evidence that major depressive disorder (MDD) is associated with initial bias against approach decisions as well as reduced sensitivity to reward magnitude on evidence accumulation [12]. Additionally, reinforcement learning (RL) models have been applied to approach avoidance, finding that reward and threat interact to generate decisions and that individual preferences weight the relative importance of reward and threat [13,14]. Finally, a Markov decision process (MDP) model under the active inference framework was used to analyze an approach-avoidance task in which individuals moved along a virtual one-dimensional runway in which position corresponded to probabilities of winning points and of exposure to affective stimuli. An emotional conflict parameter was associated with self-reported anxiety during the task [15]. Individuals with depression, anxiety, and substance use disorders displayed greater decision uncertainty, and those with substance use disorders additionally displayed lower emotional conflict [15]. Elevations in decision uncertainty in patient groups was stable over time [16]. However, there has been a lack of computational modeling of complex approach-avoidance decisions with more than two behavioral options.

Complex decisions with multiple alternatives are often modeled using a softmax decision rule in which options with higher expected value (or expected utility) are more likely to be chosen, while incorporating a degree of decisional randomness controlled by an inverse decision temperature parameter [17]. Individuals’ decisions can therefore be used to make inferences about the implicit subjective utility of different actions. Such an approach would enable a behavioral examination of the influence of both foraging opportunities and predator avoidance on the implicit utility of various potential movements, and an examination of the effect of predator proximity on this tradeoff. A stronger motivation to move away from a predator when the predator is near accords with the more general phenomenon of diminishing marginal utility (or diminishing returns), which is incorporated into models such as prospect theory [18]. In particular, the marginal utility of an additional unit of distance away from a predator may be much higher when the predator is near than when the predator is far away.

Here, we present results from the Weighing Opportunity and Loss in Foraging (WOLF) task, a new behavioral foraging task in a two-dimensional environment. This task enables a more nuanced examination of the interplay between approach and avoidance motivation in driving behavior, and specifically the effect of predator proximity in modulating avoidance motivation. We applied a novel computational model implementing a softmax decision rule, with subjective utility of movements being influenced both by approach (richness of a field for foraging) and avoidance (distance from a predator wolf) motivations. We hypothesized that human behavior could be approximated by a model in which the marginal utility of a movement away from the predator diminished with predator distance. We also hypothesized that individually estimated parameters from this computational model would predict overall task performance, mediated by aggregate measures including average field richness at each turn (for the approach-related parameter) and the number of deaths during the task (for the avoidance-related parameters).

## Materials and methods

### Participants

Forty participants (*n* = 19 women) from the San Diego area completed the WOLF task (Mean age = 27.9, *SD* = 10.69; range = 18–58; 37.5% Asian, 45% White, 2.5% multiracial, 15% prefer not to say/other; 25% Hispanic, 75% not Hispanic; Mean years of education = 15.725, *SD* = 2.85). In addition to performing the experimental task (described below), participants completed the Mood and Anxiety Symptom Questionnaire (MASQ), an assessment of affect with anxiety-specific, depression-specific, and shared symptom factors [19]. Informed consent was obtained from all participants prior to participating, and participants were monetarily compensated for their time. The institutional review board of the University of California San Diego approved all study procedures.

### WOLF task

The interactive task was displayed using PsychoPy3 [20,21] on a desktop PC where participants controlled a farmer icon within a 5 × 5 grid of resource fields with the arrow keys on a keyboard. Each field was represented by a circle that varied in color from green to yellow, indicating the resource availability at that location. The objective of the task was to maximize harvested resources while avoiding a wolf, represented by a separate icon that moved concurrently with the farmer. The task consisted of 18 games, each comprising up to 25 turns. Each game was defined by a specified field layout, wolf starting corner, and movement condition. The order of games was randomized across participants.

At the start of each game, the farmer started at the middle of the screen. Each turn lasted one second, during which the participant selected one of five possible actions: remain stationary or move up, down, left, or right. The fields were harvested automatically at the end of each decision. Harvesting was denoted in real-time by a change in field color at the end of each decision (i.e., change from green to yellow), decreasing in value by 30% per decision until depletion. Some games began with all fields in a fully green, high-resource state, while others presented spatially structured distributions, with one region of the grid containing higher-value fields and another region containing lower-value fields in a gradient reduction (see Fig 1).

**Fig 1:**
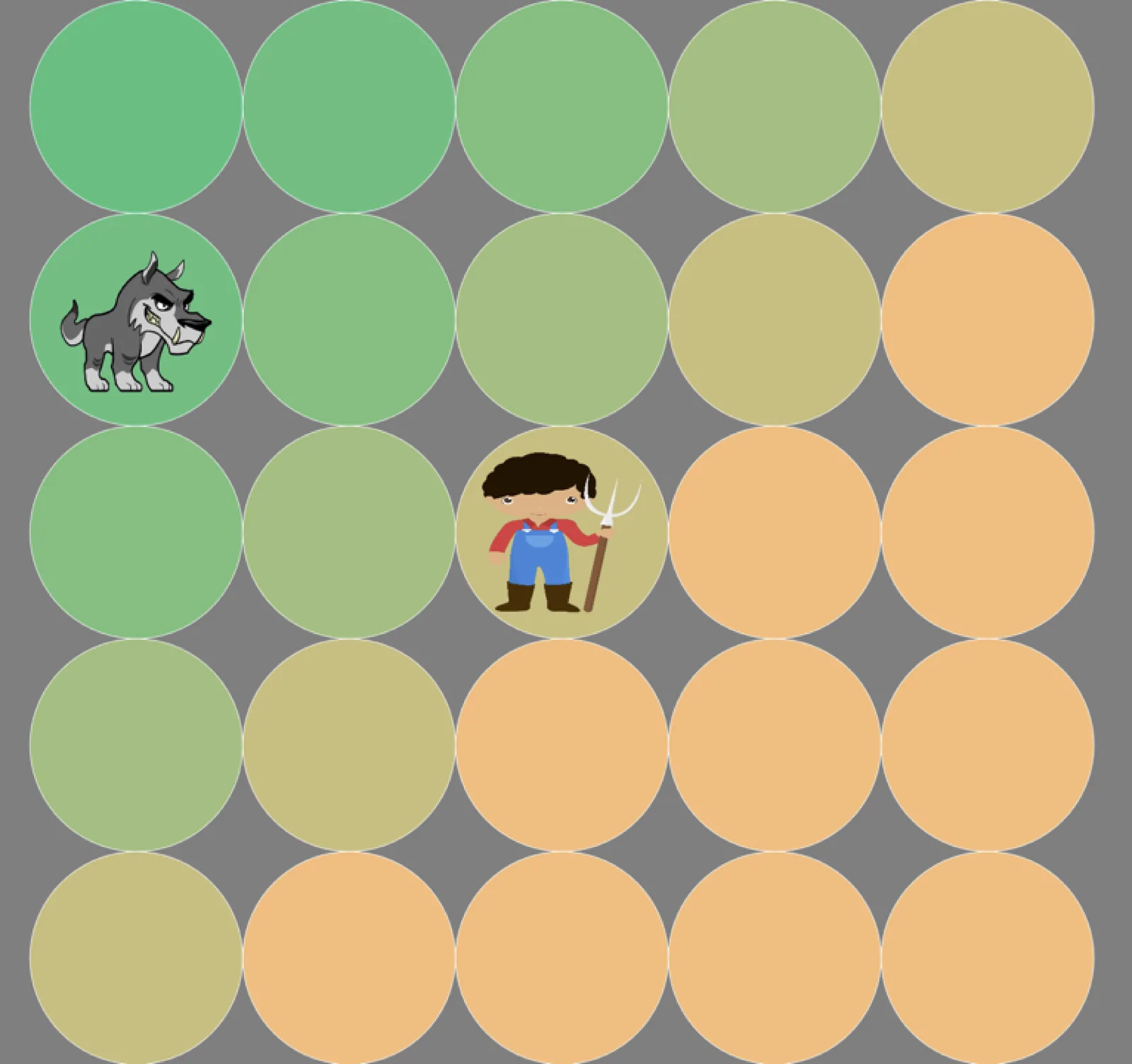
The WOLF Task. Participants controlled a farmer icon through a 5x5 grid of resource fields while under threat of predation from a wolf. On each turn, participants chose to remain stationary or move in one of four directions. The task consisted of 18 games which lasted up to 25 turns each. The wolf moved concurrently with the farmer, and participants lost all points gained during a turn if captured by the wolf.

A wolf was also present in each game, starting in a corner. The wolf moved simultaneously with the farmer according to one of three behavioral movement conditions. In random movement games (8 games), the wolf selected a movement direction with equal probability from the four cardinal directions. In stationary games (2 games), the wolf remained in its starting location throughout the game. In high-predation games (8 games), the wolf exhibited behavior biased toward the farmer’s location. This was achieved using a weighted probability mechanism, wherein movement options that reduced the distance between the wolf and the farmer were included in the selection pool 50 times, while all other movement options were included only once. A movement direction was then selected randomly from this weighted distribution, increasing the likelihood that the wolf would approach the farmer. For moving wolves, a new movement direction was sampled on each turn for each participant; thus, the movement rule was fixed by the game condition, but the resulting wolf trajectory was not generated in advance or shared across participants.

Participants accumulated points based on the total value of harvested resources. However, if the farmer and the wolf occupied the same field at any time, the game resulted in the farmer’s “death” and terminated immediately, and the participant forfeited all points accrued during that game. The next game then began with a new configuration. This task structure required participants to balance resource maximization with predator avoidance.

### Behavioral analysis

For each participant, we computed total score as a measure of overall performance on the task, as well as two aggregate measures. First, we computed the total number of deaths for each subject over the 18 total foraging games. Second, we computed the mean field richness value of all fields chosen by the subject during the task. To examine the relationship between these measures and task performance, we performed a linear regression model with each participant as a datapoint, total score as dependent variable, and mean field richness and deaths as predictors.

In order to assess whether encountering wolf behavior on one game influenced outcomes on the subsequent, we constructed models to test carryover effects across games. Wolf condition was coded categorically (random movement, high predation, and stationary). Field condition was also coded categorically (high-resource condition and gradient condition). We constructed models for three outcomes: total score for each game, mean field richness for each game (the mean field value associated with the participant’s recorded action across turns), and death for each game (a binary outcome variable). Linear mixed effects models were fit by maximum likelihood for total score and mean field richness, while a binomial logistic mixed effects model was fit for deaths. In the case of deaths, games with a current stationary wolf were excluded from the model as no deaths occurred in the stationary condition. For all models, fixed effects included previous wolf condition, current wolf condition, current field condition, and game order number, along with a random intercept for participant. The first game was excluded from analysis because it did not have a previous wolf condition. Hypothesis testing was performed via likelihood ratio test comparing the full model with a model omitting the effect of previous wolf condition.

### Computational model

Models were fit using Markov Chain Monte Carlo (MCMC) using the RStan interface of the Stan language [22]. We constructed two models for model comparison: a linear model and an inverse power law model. For both models, the probability of a participant choice *i* at each turn *t* during the task was modeled as depending on a subjective utility of each choice according to a softmax decision rule, with an inverse decision temperature parameter controlling the randomness of decisions:


$$\begin{array}{c} P\left(c_{i}\right)=\frac{exp\left(\kappa \;U_{i}^{t}\right)}{\sum_{j}exp(\kappa \;U_{j}^{t})\;}, \end{array}$$

where $P\left(c_{i}\right)$ is the probability of making choice *i*, κ is an inverse decision temperature parameter, and $U_{i}^{t}$ is the estimated utility of choice *i* on turn *t*. In order to enable model convergence, the inverse decision temperature parameter was estimated at the group level. In both models, subjective utility was modeled as a sum of two terms, representing the contribution of field richness and wolf distance, respectively, on the subjective utility of each action. In both models, the field richness term was modeled linearly, as the field richness at the next location represented by a given action multiplied by a *β*_field_ parameter which was estimated at the subject level (representing the degree of weight placed on field richness in influencing subjective utility).

For the linear model, the wolf distance term was also modeled in linear fashion, as the distance of the next location represented by a given action from the wolf location multiplied by a *β*_wolf_ parameter estimated at the subject level (representing the degree of weight placed on wolf distance in influencing subjective utility):


$$\begin{array}{c} U=\beta_{\text{field}}\;x_{\text{field}}\;+\beta_{\text{wolf}}\;x_{\text{wolf}}, \end{array}$$

where U is estimated utility, $x_{\text{field}}$ is the richness of the field at the chosen next location, and $x_{\text{wolf}}$ is the wolf distance at the chosen next location. In contrast, for the inverse power law model, the influence of wolf distance was modeled according to an inverse power law term:


$$\begin{array}{c} U=\beta_{\text{field}}\;x_{\text{field}}\;+\frac{a}{{\left(x_{\text{wolf}}+\;c\right)}^{\rho }}, \end{array}$$

where the *a* and *ρ* parameters are estimated at the subject level and the offset parameter *c* (which prevents division by zero) is fit at the group level. The *ρ* parameter was constrained to be positive and was modeled as an inverse logit transform of a normally distributed raw *ρ* parameter, multiplied by 5. This inverse power law term enables the marginal effect of wolf distance on subjective utility to be high when the wolf is near and lower when the wolf is far, declining with wolf distance in graded fashion.

Both models assume that reward and threat make additive, separable contributions to subjective utility and that decision-making occurs on a one step planning horizon. The linear model further assumes that utility varies linearly with distance from the wolf, while the inverse power law model assumes utility has an inverse power law relationship with wolf distance. This specific model was chosen to allow rapid relative change in utility with high proximity, analogous to hyperbolic temporal discounting in which immediate outcomes exert relatively higher effects [23].

We performed a model comparison to determine whether the linear model or the inverse power law model provided a better fit to data. Model comparison was performed using leave-one-out cross-validation [24].

### Relationship between model parameters and task performance

To determine whether model parameters predicted total performance on the task, we constructed a linear regression model in which the dependent variable was total score and *β*_field_, *a*, and raw *ρ* parameters were predictors.

To determine the relationship between model parameters and other model-free behavioral measures across participants, we constructed two linear regression models with participants as data points. In the first model, the dependent variable was mean field richness and the *β*_field_ parameter was the predictor (based on the hypothesis that the approach-related *β*_field_ parameter would be associated with mean field richness). In the second model, the dependent variable was number of deaths and the *a* and raw *ρ* parameters from the inverse power law term were predictors (based on the hypothesis that the avoidance-related *a* and raw *ρ* parameters would be associated with deaths).

To test the hypothesis that mean field richness (for the approach-related parameter) and deaths (for the avoidance-related parameters) mediated the relationship between model parameters and task performance, we performed three mediation analyses using the mediation package in R [25]. In the first analysis, we tested whether mean field richness mediated the effect of the *β*_field_ parameter on total score (including number of deaths as a covariate). In the second analysis, we tested whether number of deaths mediated the effect of the raw *ρ* parameter on total score (with mean field richness and the *a* parameter as covariates). In the third analysis, we tested whether number of deaths mediated the effect of the *a* parameter on total score (with mean field richness and the raw *ρ* parameter as covariates).

### Relationship between model parameters and affect

As an exploratory analysis, we tested the relationship between model parameters and self-reported affect. Three general linear models were constructed, with dependent variables of the raw *ρ* parameter, the *a* parameter, and the *β*_field_ parameter, respectively. For each model, predictors included the four MASQ subscales: anxious arousal, anhedonic depression, general distress-depressive symptoms, and general distress-anxious symptoms.

### Model simulations

We performed model simulations using the parameters that were estimated from data for each subject. For each turn during each foraging game, we computed the inferred subjective utility of each possible choice based on field richness and wolf distance, using the inverse power law model with individual *β*_field_, *a* and *ρ* parameters. We then sampled a simulated decision for each step from a softmax decision rule, incorporating the computed subjective utilities and using inverse decision temperature that had been estimated from data.

To assess the fit between model simulations and data, we calculated a relative flight value measure for each turn for both real and simulated data (based on both linear and inverse power law models). Relative flight value was calculated as the wolf distance for the next chosen field minus the wolf distance for the current field. This value ranged from −1–1, representing movement toward the wolf and movement away from the wolf, respectively. We then plotted mean relative flight value for each possible wolf distance (ranging from 1 to 8) for both real and simulated data.

## Results

### Behavioral analysis

Mean deaths per subjects was 4.13 (*SD* = 2.03) across the 18 games. In a linear regression model, total score on the task was negatively associated with number of deaths (standardized *β* = −.79, p < 0.001) and positively associated with mean field richness of choices (standardized *β* = .63, p < 0.001; R-squared .94).

Previous wolf condition had no significant effect on outcomes on the subsequent game in terms of total score (X^2^_2_ = 1.10, p = .58), mean field richness (X^2^_2_ = 2.03, p = .36), and deaths (X^2^_2_ = 1.93, p = .38).

### Model comparison

Model comparison favored the inverse power law model (leave-one-out information criterion (LOOIC) = 41866.5, standard error (SE = 149.7)) over the linear model (LOOIC = 42725.6, SE = 135.3). The posterior distribution median of the group-level *c* parameter was 1.73 (95% credible interval = [1.52, 1.96]) and of the group-level inverse temperature parameter was 0.19 (95% credible interval = [0.15, 0.25]). The mean of the individual level *ρ* parameter was 0.99 (*SD* = .67), the mean of the *a* parameter was −61.70 (*SD* = 18.65), and the mean of the *β*_field_ parameter was 5.40 (*SD* = 4.62; see Fig 2).

**Fig 2:**
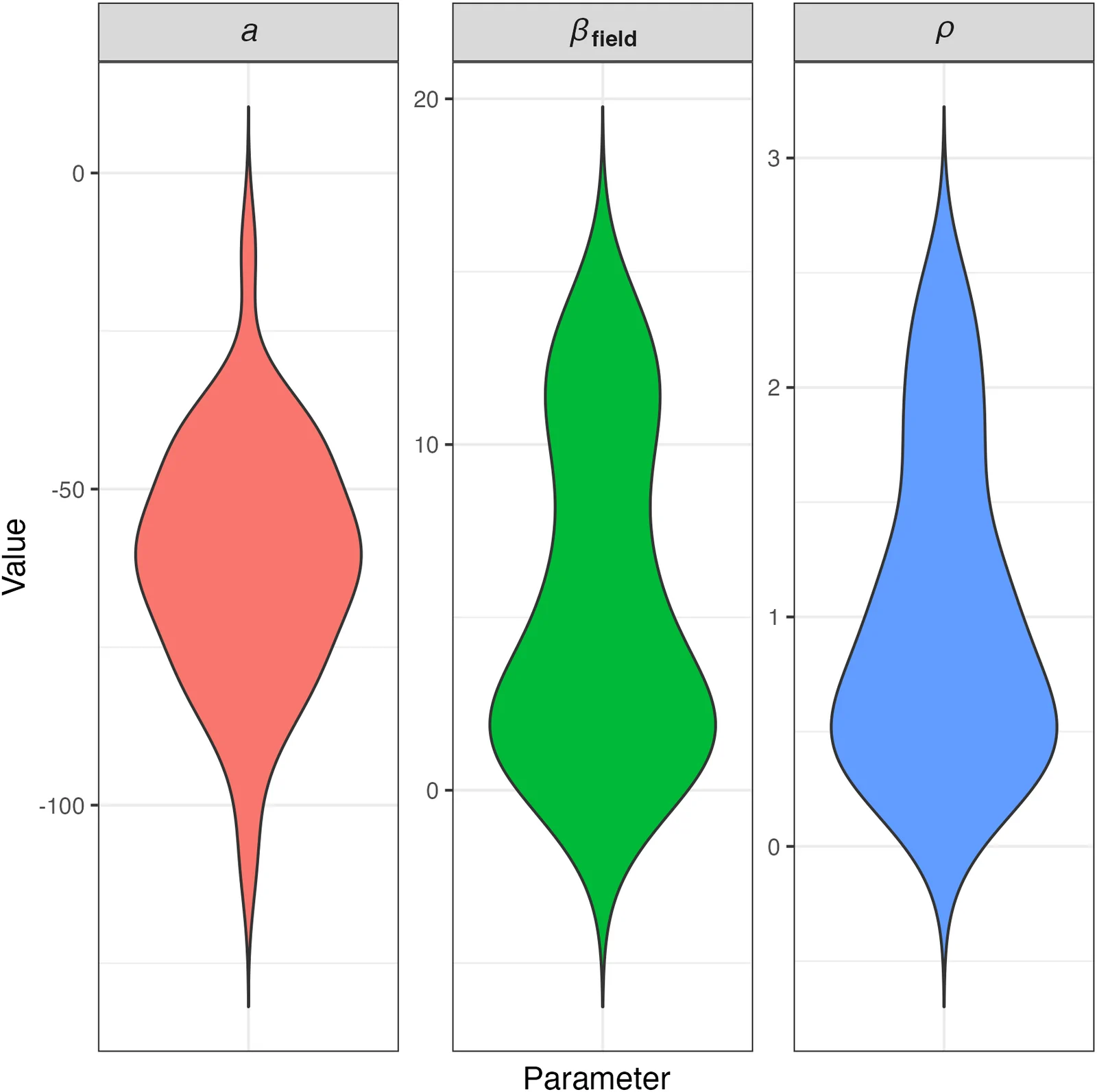
Computational Model Parameters. Violin plot showing parameters from the inverse power law model. The *a* parameter is a linear coefficient and the *ρ* parameter is the exponent in an inverse power law term which relates predator distance to avoidance motivation (implementing decreasing marginal utility of distance from the predator). The *β*_field_ parameter linearly scales the effect of field richness on the utility of a choice.

### Relationship between model parameters and task performance

In a linear regression model, total score on the task was negatively associated with the *a* parameter and positively associated with the raw *ρ* parameter and the *β*_field_ parameter (see Table 1).

**Table 1:** Linear Regressions Predicting Task Outcomes from Computational Parameters.

Predictor	Total Score		Number of Deaths		Mean Field Richness	
	*β*	*p*	*β*	*p*	*β*	*p*
***a* Parameter**	−.23	.03	.51	< .001	—	—
**Raw *ρ* Parameter**	.66	< .001	−.62	< .001	—	—
***β***_**field**_ **Parameter**	.75	< .001	—	—	.91	< .001
***R*** ^**2**^	.78		.72		.82	

Note: Values represent standardized regression coefficients. The *a* parameter represents the overall effect of the wolf on subjective utility, the *ρ* parameter controls the degree of diminishing marginal subjective utility with distance from the wolf, and he *β*_field_ parameter scales the influence of field richness on subjective utility.

In a linear regression model, number of deaths was positively associated with the *a* parameter and negatively associated with the raw *ρ* parameter (see Table 1). In another linear regression model, mean field richness of choices was positively associated with the *β*_field_ parameter (see Table 1).

Mediation analysis revealed that number of deaths fully mediated the relationship between the *a* parameter and total score (Average Causal Mediated Effect (ACME) = −0.44, 95% confidence interval (CI) = [−0.65, −0.27], p < .001; proportion mediated = 1.15, 95% CI = [0.89, 1.67], p < .001; Fig 3a). Number of deaths also fully mediated the relationship between the raw *ρ* parameter and total score (ACME = 0.49, 95% CI = [0.28, 0.68], p < 0.001; proportion mediated = 0.99, 95% CI = [0.79, 1.24], p < 0.001; Fig 3b). Finally, mean field richness of choices fully mediated the relationship between the *β*_field_ parameter and total score (ACME = 0.61, 95% CI = [0.36, 0.92], p < 0.001; proportion mediated = 1.09, 95% CI = [0.68, 1.59], p < 0.001; Fig 3c).

**Fig 3:**
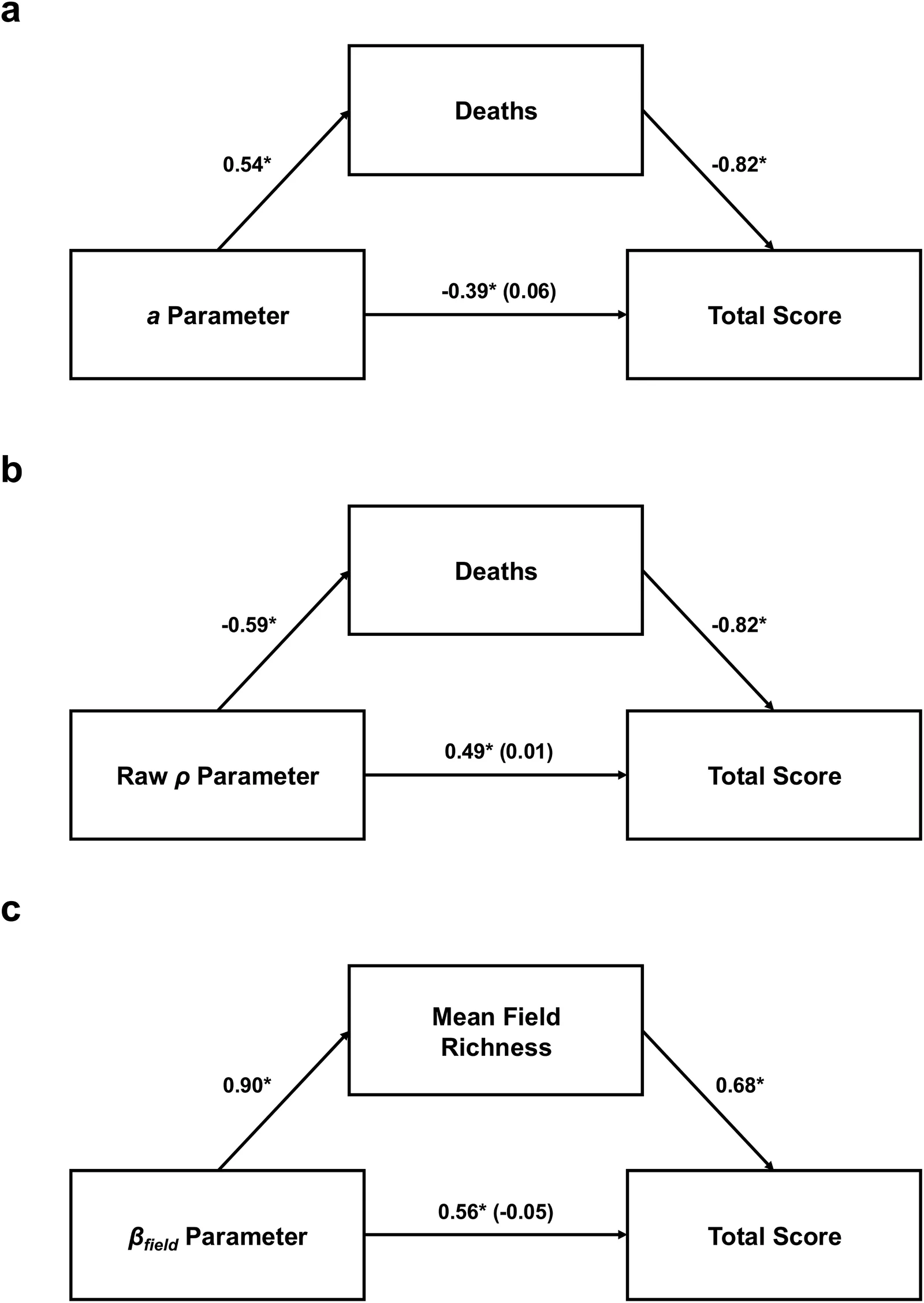
Mediation Analyses. a. Number of deaths fully mediate the relationship between the *a* parameter and total score on the task. b. Number of deaths fully mediate the relationship between the *ρ* parameter and total score on the task. c. The mean richness of fields chosen fully mediates the relationship between the *β*_*field*_ parameter and total score on the task.

### Relationship between model parameters and affect

In the exploratory analyses, the linear model predicting the raw *ρ* parameter based on MASQ subscales was significant (R-squared = .28, p = .02) and there was a significant negative association between the MASQ anxious arousal subscale and the raw *ρ* parameter (standardized *β* = −0.12, p = .009), controlling for the other MASQ subscales. The models predicting the *a* parameter and the *β*_field_ parameter based on MASQ subscales were not significant overall.

### Model simulations

In addition to providing a better model fit based on leave-one-out cross validation, the inverse power law model also provided a better fit visually when the wolf distance was low compared to the linear model when relative flight was plotted against wolf distance (see Fig 4).

**Fig 4:**
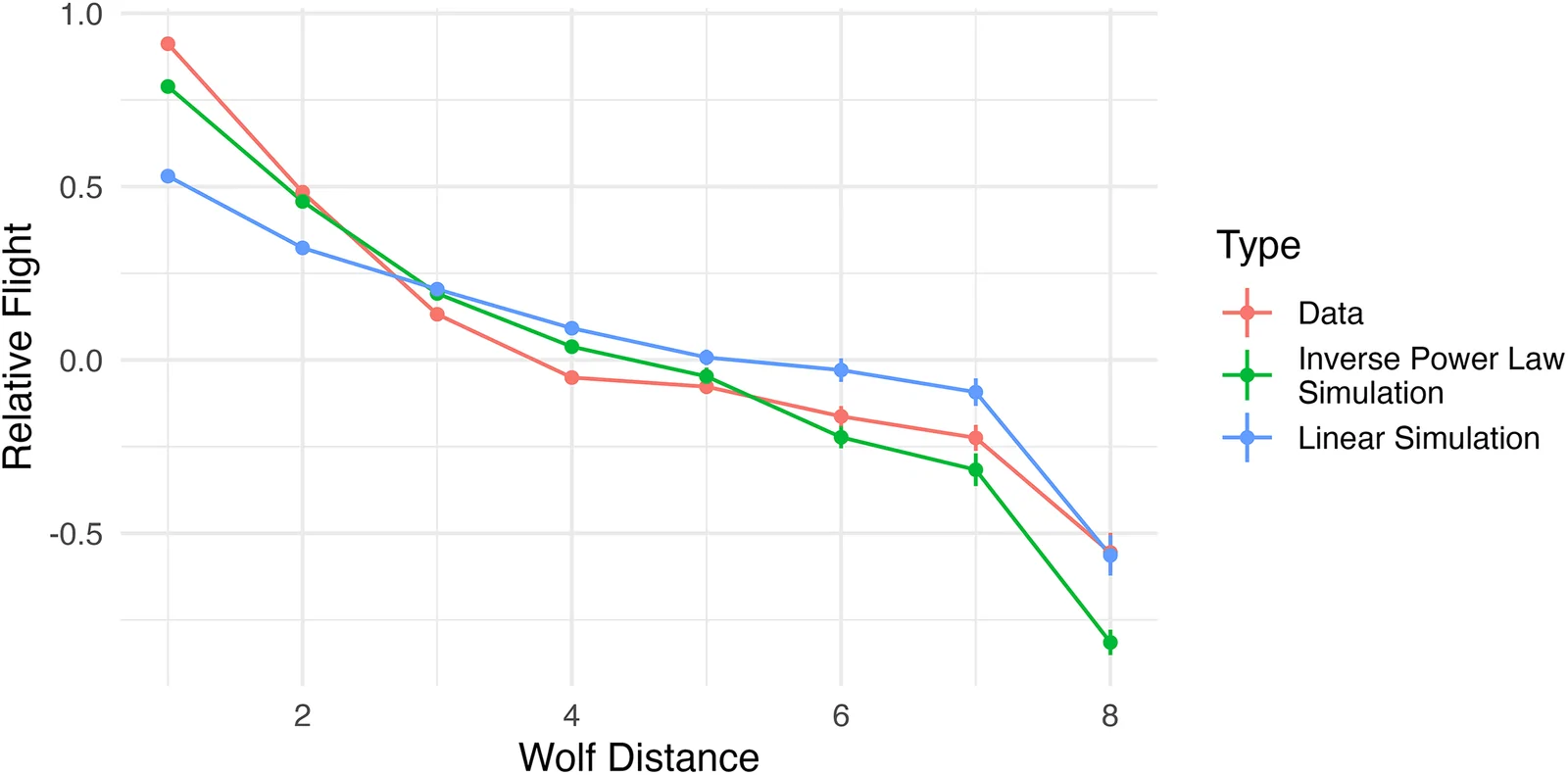
Model Simulations and Data. Simulations from two models (an inverse power law model and a linear model) compared with human data. Relative flight is calculated as the wolf distance for the next chosen field minus the wolf distance for the current field, representing movement toward the wolf and movement away from the wolf, respectively. In the linear model, the subjective utility of an action depended linearly on distance from the wolf, whereas in the inverse power law model, there was diminishing marginal subjective utility of distance from the wolf with greater distance. The inverse power law provided a better fit to data when the wolf distance was low. Error bars represent standard error across subjects.

## Discussion

In this study, we applied a computational decision-making model to examine approach-avoidance behavior in the WOLF task, a novel foraging task. Decisions were modeled as depending on the subjective utility of each action, which in turn depended on approach motivation (based on field richness for foraging) and avoidance motivation (based on distance from a wolf predator). Model comparison revealed that an inverse power law best modeled the effect of wolf distance on subjective utility, indicating that the marginal effect of wolf distance was greater when the wolf was near compared to when the wolf was far. Model parameters explained more than 75% of the variance in total score on the task, mediated by mean field richness of choices (for the approach-related parameter) and number of deaths (for the avoidance-related parameters). The *β*_field_ parameter, which scales the influence of field richness on subjective utility, was positively associated with mean richness of chosen fields and with total score. A higher (less negative) *a* parameter (the coefficient which scales the inverse power law, representing overall effect of the wolf on subjective utility) was positively associated with deaths and negatively associated with total score. A higher *ρ* parameter (the exponent in the power law, controlling the degree of diminishing marginal subjective utility with distance from the wolf) was negatively associated with deaths and positively associated with total score.

Previous research has measured flight initiation distance (FID), a key concept from the ethological foraging literature, in humans under varying conditions (e.g., different predator speeds) [3]. A classic analysis of the economics of FID assumed that the cost (i.e., negative utility) of staying (vs. fleeing) depends linearly on risk of capture by the predator, which increases as the predator nears [4]. However, this model was qualitative rather than quantitative. More recent analyses have examined the effect of a wider range of costs and benefits on optimal FID (i.e., FID that maximizes evolutionary fitness), while noting that fitness functions are uncertain and animals may use heuristics in deciding when to flee [5]. A strength of the task and modeling approach used in the present manuscript is the ability to explicitly and quantitatively model the subjective cost curve (i.e., negative utility curve) for predator distance at the individual level. This is achieved through a rich task framework in which the participant selects one of five possible movements at each step during each game (rather than making a single binary decision to flee once per game) and through application of an explicit decision model which depends on the subjective utility of each action.

Previous models of FID have considered different possible relationships between predator distance and cost, including a linear relationship and a curved relationship in which the curve is steeper when the predator is nearest [5]. The present study found that the best fit was achieved by a model in which the curve was steepest when the predator was near, i.e., that the marginal subjective utility of distance from the predator decreased with increasing predator distance. Diminishing marginal utility (instantiated in a concave utility function) is a ubiquitous phenomenon across a wide range of economic situations [26] and was first articulated more than a century ago [27]. It can be observed in experimental behavioral paradigms and is related to individual differences in risk preferences [28,29]. A concave utility function for resources such as food has been analyzed in terms of optimality in maximizing the probability of survival [30]. Mapping the utility curve for safety from threats such as predation (as opposed to the curve for appetitive stimuli such as food) may have important implications for individual differences in avoidance behavior and psychiatric disorders such as anxiety disorders.

The concept of FID assumes a binary distinction between continued foraging and a decision to flee, which may not be applicable to all approach-avoidance scenarios in foraging-like contexts. Instead, there may be a complex interplay between approach and avoidance motivation in selecting from a range of possible actions at each timepoint. The WOLF task represents a rich framework for analyzing these more complex decision scenarios. While the current modeling approach is heuristic (using an inverse power law curve rather than an explicit model of probability of capture by the predator) and greedy (i.e., only considering the field richness and predator distance one turn in the future), there is potential for future work to implement more complex multi-turn planning models which can incorporate complex spatial structure of resources (field richness) and models of predator behavior. Despite the relative simplicity of the current modeling approach, the model parameters explained a substantial percentage of the variance in performance on the task, indicating that the model was accurate in predicting individual differences in task behavior.

Our exploratory finding that the raw *ρ* parameter was negatively associated with anxious arousal suggests that individual affective differences may influence the modulation of avoidance motivation by distance from threat. In particular, a lower raw *ρ* parameter corresponds to a more linear relationship between predator distance and avoidance motivation, rather than a steep relationship for a nearby predator that quickly diminishes with distance. This suggests that individuals with higher arousal may perceive threats as persistently salient even at greater distance rather than strongly modulating threat salience based on threat proximity. However, given the relatively low sample size and the exploratory nature of this finding, it will be important to replicate in future samples. Future research can more fully examine the role of affect in individual differences in approach-avoidance behavior on the WOLF task. Future research can also examine the relationship between foraging behavior and fluctuations in affect within individuals, given previous evidence that individuals respond to task-evoked stress in a foraging context by increasing both foraging and hiding behaviors [31].

Future research will be required to assess the generalizability of the findings. It will be important to determine whether the relationship between affect and foraging parameters extend to clinical populations. Additionally, it is important to note that foraging for virtual points in the presence of a virtual predator is unlikely to fully capture the mechanisms decision-making under physical threat. Real world decision-making can differ in other respects, such as involving uncertainty about the current state of the environment. Another important direction for future research is to combine the WOLF task with measures of neural activity (such as functional magnetic resonance imaging) or other biological measures. Previous research has implicated a variety of neural regions in approach-avoidance paradigms including prefrontal cortex, insula, striatum, hippocampus, amygdala, anterior cingulate cortex, and periaqueductal gray [2,9,32,33]. The WOLF task may enable further examination of the effect of these circuits on behavior in a rich decision context, and their relationship to the subjective utility curve. These brain-behavior relationships may also have implications for individual differences in fear and anxiety and for clinical disorders such as anxiety disorders and posttraumatic stress disorder. For example, anxiety may involve over-representation of avoidance related to limbic overactivation, under-representation of approach valuation due to altered striatal function, and difficulty integrating approach and avoidance representations [7]. In addition, PTSD may involve sacrifice of rewards to avoid threats [34]. Examining the curved relationship between neural activation and distance from threat on the WOLF task may shed additional light on the neural underpinnings of avoidance behaviors in these disorders, especially given the potential finding of a relationship between affect and WOLF task behavior. Future research in larger samples will enable fuller examination of the relationship between model parameters and mood, anxiety, and psychiatric disorders.

In conclusion, approach-avoidance behavior in a two-dimensional foraging context under threat of predation can be modeled based on the subjective utility of each possible action, which in turn depends on approach motivation (richness of resources for foraging) and avoidance motivation (distance from a predator). We explicitly model the dependence of subjective utility on predator proximity and demonstrate diminishing subjective utility of distance from a predator. Future work can extend these results by examining neural correlates of foraging in a two-dimensional context and relationship to neural activity. Foraging paradigms with rich structure offer promise to improve understanding of approach-avoidance behavior in health and in mental health disorders.

## References

[1] Schneirla TC. An evolutionary and developmental theory of biphasic processes underlying approach and withdrawal. Nebraska symposium on motivation. Oxford, England: Univer. Nebraska Press; 1959. p. 1–42.

[2] Aupperle RL, Melrose AJ, Francisco A, Paulus MP, Stein MB. Neural substrates of approach-avoidance conflict decision-making. Hum Brain Mapp. 2015;36(2):449–62. doi: 10.1002/hbm.22639 25224633 PMC4300249

[3] Mobbs D, Trimmer PC, Blumstein DT, Dayan P. Foraging for foundations in decision neuroscience: Insights from ethology. Nat Rev Neurosci. 2018;19(7):419–27. doi: 10.1038/s41583-018-0010-7 29752468 PMC6786488

[4] Ydenberg RC, Dill LM. The economics of fleeing from predators. Advances in the Study of Behavior. Elsevier. 1986. p. 229–49. doi: 10.1016/s0065-3454(08)60192-8

[5] Cooper WE, Frederick WG. Optimal flight initiation distance. J Theor Biol. 2007;244(1):59–67. doi: 10.1016/j.jtbi.2006.07.011 16949619

[6] Bomyea J, Choi SH, Sweet A, Stein M, Paulus M, Taylor C. Neural changes in reward processing following approach avoidance training for depression. Soc Cogn Affect Neurosci. 2021;17(3):336–49. doi: 10.1093/scan/nsab107 34643736 PMC8881638

[7] Aupperle RL, Paulus MP. Neural systems underlying approach and avoidance in anxiety disorders. Dialogues Clin Neurosci. 2010;12(4):517–31. doi: 10.31887/DCNS.2010.12.4/raupperle 21319496 PMC3181993

[8] Harlé KM, Simmons AN, Bomyea J, Spadoni AD, Taylor CT. Higher affective congruency in the approach-avoidance task is associated with insular deactivation to dynamic facial expressions. Neuropsychologia. 2021;151:107734. doi: 10.1016/j.neuropsychologia.2020.107734 33358774 PMC7856231

[9] Mobbs D, Petrovic P, Marchant JL, Hassabis D, Weiskopf N, Seymour B, et al. When fear is near: Threat imminence elicits prefrontal-periaqueductal gray shifts in humans. Science. 2007;317(5841):1079–83. doi: 10.1126/science.1144298 17717184 PMC2648508

[10] Qi S, Hassabis D, Sun J, Guo F, Daw N, Mobbs D. How cognitive and reactive fear circuits optimize escape decisions in humans. Proc Natl Acad Sci U S A. 2018;115(12):3186–91. doi: 10.1073/pnas.1712314115 29507207 PMC5866541

[11] Letkiewicz AM, Kottler HC, Shankman SA, Cochran AL. Quantifying aberrant approach-avoidance conflict in psychopathology: A review of computational approaches. Neurosci Biobehav Rev. 2023;147:105103. doi: 10.1016/j.neubiorev.2023.105103 36804398 PMC10023482

[12] Pedersen ML, Ironside M, Amemori K-I, McGrath CL, Kang MS, Graybiel AM, et al. Computational phenotyping of brain-behavior dynamics underlying approach-avoidance conflict in major depressive disorder. PLoS Comput Biol. 2021;17(5):e1008955. doi: 10.1371/journal.pcbi.1008955 33970903 PMC8136861

[13] Talmi D, Dayan P, Kiebel SJ, Frith CD, Dolan RJ. How humans integrate the prospects of pain and reward during choice. J Neurosci. 2009;29(46):14617–26. doi: 10.1523/JNEUROSCI.2026-09.2009 19923294 PMC2948535

[14] Moughrabi N, Botsford C, Gruichich TS, Azar A, Heilicher M, Hiser J, et al. Large-scale neural network computations and multivariate representations during approach-avoidance conflict decision-making. Neuroimage. 2022;264:119709. doi: 10.1016/j.neuroimage.2022.119709 36283543 PMC9835092

[15] Smith R, Kirlic N, Stewart JL, Touthang J, Kuplicki R, Khalsa SS, et al. Greater decision uncertainty characterizes a transdiagnostic patient sample during approach-avoidance conflict: A computational modelling approach. J Psychiatry Neurosci. 2021;46(1):E74–87. doi: 10.1503/jpn.200032 33119490 PMC7955838

[16] Smith R, Kirlic N, Stewart JL, Touthang J, Kuplicki R, McDermott TJ, et al. Long-term stability of computational parameters during approach-avoidance conflict in a transdiagnostic psychiatric patient sample. Sci Rep. 2021;11(1):11783. doi: 10.1038/s41598-021-91308-x 34083701 PMC8175390

[17] Sutton RS, Barto AG. Reinforcement Learning: An Introduction. Cambridge: MIT Press; 1998.

[18] Tversky A, Kahneman D. Advances in prospect theory: Cumulative representation of uncertainty. J Risk Uncertainty. 1992;5(4):297–323. doi: 10.1007/bf00122574

[19] Watson D, Clark LA. Mood and anxiety symptom questionnaire. 1991. doi: 10.1037/t13679-000

[20] Peirce JW. PsychoPy--Psychophysics software in Python. J Neurosci Methods. 2007;162(1–2):8–13. doi: 10.1016/j.jneumeth.2006.11.017 17254636 PMC2018741

[21] Peirce J, Gray JR, Simpson S, MacAskill M, Höchenberger R, Sogo H, et al. PsychoPy2: Experiments in behavior made easy. Behav Res Methods. 2019;51(1):195–203. doi: 10.3758/s13428-018-01193-y 30734206 PMC6420413

[22] Stan Development Team. RStan: the R interface to Stan. 2018.

[23] Myerson J, Green L. Discounting of delayed rewards: Models of individual choice. J Exp Anal Behav. 1995;64(3):263–76. doi: 10.1901/jeab.1995.64-263 16812772 PMC1350137

[24] Vehtari A, Gelman A, Gabry J. Practical Bayesian model evaluation using leave-one-out cross-validation and WAIC. Stat Comput. 2017;27(5). doi: 10.1007/s11222-016-9696-4

[25] Tingley D, Yamamoto T, Hirose K, Keele L, Imai K. Mediation: R package for causal mediation analysis. J Stat Softw. 2014;59(5). doi: 10.18637/jss.v059.i05

[26] Kimball MS, Reck D, Zhang F, Ohtake F, Tsutsui Y. Diminishing marginal utility revisited. 2024.

[27] Ormazabal KM. The law of diminishing marginal utility in alfred marshall’s principles of economics. The European Journal of the History of Economic Thought. 1995;2(1):91–126. doi: 10.1080/10427719500000096

[28] Howlett JR, Paulus MP. Individual differences in subjective utility and risk preferences: The influence of hedonic capacity and trait anxiety. Front Psychiatry. 2017;8:88. doi: 10.3389/fpsyt.2017.00088 28588508 PMC5438983

[29] Howlett JR, Paulus MP. A parallel accumulator model accounts for decision randomness when deciding on risky prospects with different expected value. PLoS One. 2020;15(7):e0233761. doi: 10.1371/journal.pone.0233761 32702026 PMC7377428

[30] McDermott R, Fowler JH, Smirnov O. On the evolutionary origin of prospect theory preferences. J Polit. 2008;70(2):335–50. doi: 10.1017/S0022381608080341

[31] Trier HA, O’Reilly JX, Spiering L, Yishan SM, Kolling N, Rushworth MFS, et al. Emotions and individual differences shape human foraging under threat. Nat Ment Health. 2025;3(4):444–65. doi: 10.1038/s44220-025-00393-8 40213470 PMC11978516

[32] Bach DR, Guitart-Masip M, Packard PA, Miró J, Falip M, Fuentemilla L, et al. Human hippocampus arbitrates approach-avoidance conflict. Curr Biol. 2014;24(5):541–7. doi: 10.1016/j.cub.2014.01.046 24560572 PMC3969259

[33] Gold AL, Morey RA, McCarthy G. Amygdala-prefrontal cortex functional connectivity during threat-induced anxiety and goal distraction. Biol Psychiatry. 2015;77(4):394–403. doi: 10.1016/j.biopsych.2014.03.030 24882566 PMC4349396

[34] Weaver SS, Kroska EB, Ross MC, Sartin-Tarm A, Sellnow KA, Schaumberg K, et al. Sacrificing reward to avoid threat: Characterizing PTSD in the context of a trauma-related approach-avoidance conflict task. J Abnorm Psychol. 2020;129(5):457–68. doi: 10.1037/abn0000528 32437204 PMC7393639

